# Inhibition of Donor-Reactive CD8+ T Cell Responses by Selective CD28 Blockade Is Independent of Reduced ICOS Expression

**DOI:** 10.1371/journal.pone.0130490

**Published:** 2015-06-22

**Authors:** Danya Liu, Suzanne J. Suchard, Steve G. Nadler, Mandy L. Ford

**Affiliations:** 1 Emory Transplant Center and Department of Surgery, Emory University, Atlanta, GA 30322, United States of America; 2 Bristol-Myers Squibb Company, Princeton, NJ, United States of America; University of Toledo, UNITED STATES

## Abstract

Programmed T cell differentiation is critically influenced by the complement of costimulatory and coinhibitory signals transmitted during initial antigen encounter. We previously showed that selective CD28 blockade with novel domain antibodies that leave CTLA-4-mediated coinhibitory signaling intact resulted in more profound attenuation of donor-reactive T cell responses and improved graft survival in a murine transplant model. Selective CD28 blockade was also associated with decreased ICOS expression on donor-reactive CD8^+^ T cell responses as compared to CTLA-4 Ig, but the functional importance of this reduced ICOS expression was not known. In this study, we created retrogenic donor-reactive CD8^+^ T cells that overexpress ICOS in order to determine whether reduced ICOS expression mechanistically underlies the increased efficacy of selective CD28 blockade in controlling graft-specific T cell responses as compared to conventional costimulation blockade with CTLA-4 Ig. Results indicated that the ability of selective CD28 blockade to blunt donor-reactive CD8^+^ T cell expansion following transplantation was independent of its ability to inhibit ICOS expression. Furthermore, we have previously published that 2B4 coinhibitory signals are functionally important for controlling graft-specific CD8^+^ T cell responses in mice treated with CD28 blockade. Here we used a co-adoptive transfer approach to determine that 2B4 coinhibitory signals on antigen-specific CD8^+^ T cells function in a cell-intrinsic manner to limit ICOS expression in the setting of selective CD28 blockade.

## Introduction

T cell activation is triggered following TCR recognition of cognate antigen/MHC complexes, but the ensuing programmed differentiation is profoundly modified by the complement of costimulatory and coinhibitory signals transmitted during initial antigen encounter [1, 2]. It is increasingly recognized that the initial cosignals perceived during T cell activation result in transcription and translation of “secondary” inducible costimulatory or coinhibitory molecules, resulting in further fine-tuning of the response. This multi-tiered process of T cell costimulation ensures that the appropriate T cell differentiation program is initiated and is exquisitely well suited to the microenvironment in which the T cell was primed. As such, pharmacologic manipulation of T cell cosignaling pathways represents an attractive target for therapeutic intervention in a host of immune-mediated diseases, including autoimmunity, transplant rejection, and cancer [2].

The hallmark T cell costimulatory molecule is CD28, a constitutively expressed cell surface protein that likely represents the “first line” of T cell costimulatory signals received following APC encounter [3]. Given its functional importance in the initiation of T cell expansion and differentiation, CD28 has been an attractive target for therapeutic intervention [4], and blockers of the CD28 pathway are now approved for use in autoimmunity (abatacept) and transplantation (belatacept). ICOS (inducible T cell costimulator) is a member of the CD28 family of cosignaling molecules [5], but unlike CD28 ICOS is not expressed on resting CD4^+^ or CD8^+^ T cells but is dynamically regulated during the course of T cell activation [6]. Following upregulation and encounter of its ligand B7-h1 (ICOS-L), ICOS delivers additional co-stimulatory signals to further enhance T-cell activation and differentiation into cytokine-producing effector cells [6, 7].

Models of autoimmunity revealed that ICOS signaling is critical for T cell-mediated pathogenicity in experimental autoimmune encephalomyelitis and the development of type 1 diabetes [8], and that ICOS blockade could be efficacious in treating on-going activated T cell responses and reversing autoimmunity during active disease [9, 10]. Similarly, research in experimental transplant models have demonstrated that costimulation through ICOS is required for the development of both acute and chronic rejection [11, 12]. In a recent study, ICOS antagonism synergized with CTLA-4-Ig to inhibit the effector function of donor-reactive memory T cells and prolong graft survival [13].

While blockade of ICOS signals continues to be investigated in experimental and pre-clinical models, as mentioned above blockade of the CD28 pathway has reached clinical application in that the CTLA-4 Ig fusion proteins abatacept and belatacept are currently approved for use in autoimmunity and transplantation, respectively. However, these CTLA-4 Ig fusion proteins bind the CD80 and CD86 ligands and thus block CD28 costimulatory signals, but also inhibit CTLA-4 mediated coinhibitory signals [14]. Thus, we have utilized selective CD28 blockade using a novel CD28-specific domain antibody in order to more specifically inhibit CD28 mediated costimulatory signals while leaving physiologically important CTLA-4 coinhibitory signals intact. Our recent report indicated that indeed selective CD28 blockade showed increased efficacy in inhibiting alloreactive CD8^+^ T cell responses and prolonging allograft survival [15]. In order to determine the mechanism underlying the more profound inhibition of donor-reactive CD8^+^ T cell responses following treatment with the anti-CD28 dAb as compared to CTLA-4 Ig, we examined the phenotype of donor-reactive CD8^+^ T cells under both treatment conditions, and observed two important differences.

First, we observed that while CTLA-4 Ig treatment resulted in only a modest decline in the expression of the inducible costimulatory molecule ICOS, treatment with anti-CD28dAb resulted in a significant diminution of its expression on both CD4^+^ and CD8^+^ donor-reactive T cells [15]. Thus, our previous study identified an association of decreased ICOS expression with increased control of donor-reactive CD8^+^ T cell responses and improved graft survival, but the functional importance of this ICOS downregulation is not known. Second, CD8^+^ T cells from mice treated with anti-CD28dAb exhibited a significant and selective increase in the expression of the coinhibitory receptor 2B4 (CD244), a member of the CD2 family best known for its expression on NK cells and also associated with CD8^+^ T cell exhaustion [16–18]. However, the mechanisms underlying the regulation of 2B4 expression are unknown, and we hypothesized that decreased ICOS expression observed following selective CD28 blockade might induce the subsequent upregulation of 2B4. Thus, in this study, we created retrogenic donor-reactive CD8^+^ T cells that overexpress ICOS in order to determine whether reduced ICOS expression mechanistically underlies the increased efficacy in controlling graft-specific T cell responses observed with selective CD28 blockade as compared to conventional costimulation blockade with CTLA-4 Ig.

## Materials and Methods

### Mice

C57BL/6 (H-2b) and BALB/c (H-2d) mice were obtained from the National Cancer Institute (Frederick, MD). OT-I [19] and OT-II [20] transgenic mice, purchased from Taconic Farms (Germantown, NY), were bred to Thy1.1^+^ background at Emory University. mOVA mice (C57BL/6 background, H-2b) [21] were a gift from Dr. Marc Jenkins (University of Minnesota, Minneapolis, MN). 2B4 (CD244)^-/-^ animals on a B6 background [18] were a gift of Dr. Cox Terhorst (Beth Israel Deaconess Medical Center, Harvard Medical School, Boston, MA), and were bred onto OT-I x Thy1.1 background at Emory University. This study was carried out in strict accordance with the recommendations in the Guide for the Care and Use of Laboratory Animals. The protocol was approved by the Institutional Animal Care and Use Committee of Emory University (protocol number: DAR-2002050-092815GN). All surgery was performed under general anesthesia, and all efforts were made to minimize suffering. All animals were housed in pathogen-free animal facilities at Emory University.

### Donor-Reactive T Cell Adoptive Transfers

For adoptive transfers of donor-reactive T cells, spleen and mesenteric LNs isolated from Thy1.1^+^ OT-I and Thy1.1^+^ OT-II mice were processed and stained with monoclonal antibodies for CD4 and CD8 (both from Invitrogen), Thy1.1, and Vα2 (BD Pharmingen) for flow cytometry analysis. Cells were resuspended in PBS and 1.0x106 of each Thy1.1^+^ OT-I and OT-II were injected i.v. 24–48 h prior to skin transplantation.

### Skin Transplantation and In Vivo Costimulatory Molecule Blockade

Full thickness tail and ear skins were transplanted onto the dorsal thorax of recipient mice and secured with adhesive bandages as previously described [22]. Where indicated, mice were injected with 100 μg control Vκ dAb, 100 μg anti-CD28 dAb or 250 μg CTLA-4 Ig (all Bristol-Myers Squibb, Princeton, NJ) on days 0, 2, 4, 6, and three times per week continuously thereafter until the mice were sacrificed or until day 50 (for skin graft survival experiments).

### ICOS Plasmid Construction and Transfection

The murine *Icos* gene was derived from mouse cDNA (Thermo, Cat. MMM1013-7510186) and produced by PCR using the primers: (Forward primer: gcGAATTCgccacc ATG AAG CCG TAC TTC TGC CGT GTC TTT G, Reverse primer: cgCTCGAG TTA TGA GGT CAC ACC TGC AAG). The resulting PCR fragment was cloned into the pMY-IRES-GFP retroviral vector (Cell Biolabs, RV-021) using EcoRI and XhoI cut sites. The Platinum-E retroviral packaging cell line (Cell Biolabs, RV-101. Ecotropic for rat and mouse cells) was used to produce the ICOS-containing retrovirus. The cells were maintained in Dulbecco's modified Eagle's medium (DMEM) supplemented with 10% heat-inactivated fetal calf serum (FCS), 1ug/ml puromycin, 10ug/ml blasticidine, 100 U/mL penicillin, and 100 μg/mL streptomycin at 37°C in a 5% CO2, humidified atmosphere. The packaging cells were incubated in 10-cm plates at 4.5 × 106/plate overnight at 37°C. Transfections were performed by the reagent Lipofectamine LTX (Invitrogen, 15338–100). Cells were transiently transfected with 10 μg DNA (ICOS plasmid DNA or empty vector control). After 48 hours incubation the culture supernatant was harvested and virus was concentrated per manufacturer’s instructions (Cell Biolabs, RV-201).

### Retroviral transduction and generation of ICOSrg OT-I T cells

Two days before transduction, bone marrow cells were harvested from 8 to 12 week old OT-I transgenic mice and cultured at 1.5X107 cells per 10 cm plate in 15 ml DMEM supplemented with 15% heat-inactivated fetal calf serum (FCS), 100 U/mL penicillin, and 100 μg/mL streptomycin, 10mM hepes, 20 ng/ml murine interleukin-3 (IL-3), 50 ng/ml human IL-6 and 50 ng/ml murine stem cell factor (SCF) (R&D Systems). The concentrated virus was transduced into the pre-cultured BMCs. After 48 hours incubation bone marrow cells were collected and washed. Sub lethally irradiated (800 rads) WT B6 recipients were injected IV with 4 × 106 bone marrow cells in PBS. Splenocytes from these BM chimeras were harvested 6–8 weeks post-transplant and were enriched by negative selection using a CD8a^+^ T cell Isolation Kit II (Miltenyi Biotec,). Purity of CD8a^+^ T cells was over 80%. Cells were then stained with anti-CD8 Pac Orange, anti-Thy1.1 PerCP, and anti-ICOS APC and CD8^+^ Thy1.1^+^ ICOS^+^ cells were purified by FACS sorting on a BD FACS Aria. Post-sort ICOS-OT-I T cell populations were over 95% pure.

### Flow Cytometry and Intracellular Cytokine Staining

Spleens or graft-draining axillary and brachial LNs were stained for CD4 and CD8 (both from Invitrogen) and Thy1.1 (BD Pharmingen). For phenotypic analysis cells were also surface-stained with anti-ICOS, anti-2B4, anti-PD-1, anti-KLRG-1, anti-CD127, and anti-BTLA, anti- Eomesodermin, and anti-BLIMP-1 (all Pharmingen). CTLA-4 expression was measured intracellularly using an intracellular staining kit (BD Pharmingen) following ex vivo restimulation. Absolute numbers were calculated using TruCount bead analysis according to the manufacturer’s instructions. Samples were analyzed on an LSRII flow cytometer (BD Biosciences). Data was analyzed using FlowJo software (Treestar, San Carlos, CA). For intracellular cytokine staining, splenocytes were stimulated with 10 nM OVA257-264 (SIINFEKL) or 10 μM OVA323-339 (ISQAVHAAHAEINEAGR) (Genscript, Inc.) where indicated, in the presence of 10 μg/mL Brefeldin A for 4 hours. An intracellular staining kit was used to detect TNF and IFN-γ (all from BD Pharmingen), according to manufacturer’s instructions.

### Statistical Analysis

Survival data were plotted on Kaplan-Meier curves and log-rank tests were performed. For analysis of T cell responses, non-parametric Mann-Whitney U-tests were performed. Results were considered significant if p<0.05. All analyses were done using GraphPad Prism software (GraphPad Software Inc.). In all legends and figures, *p<0.05, **p<0.01, ***p<0.001.

## Results

### Reduced ICOS expression following selective CD28 blockade is CTLA-4 dependent

**Fig 1:**
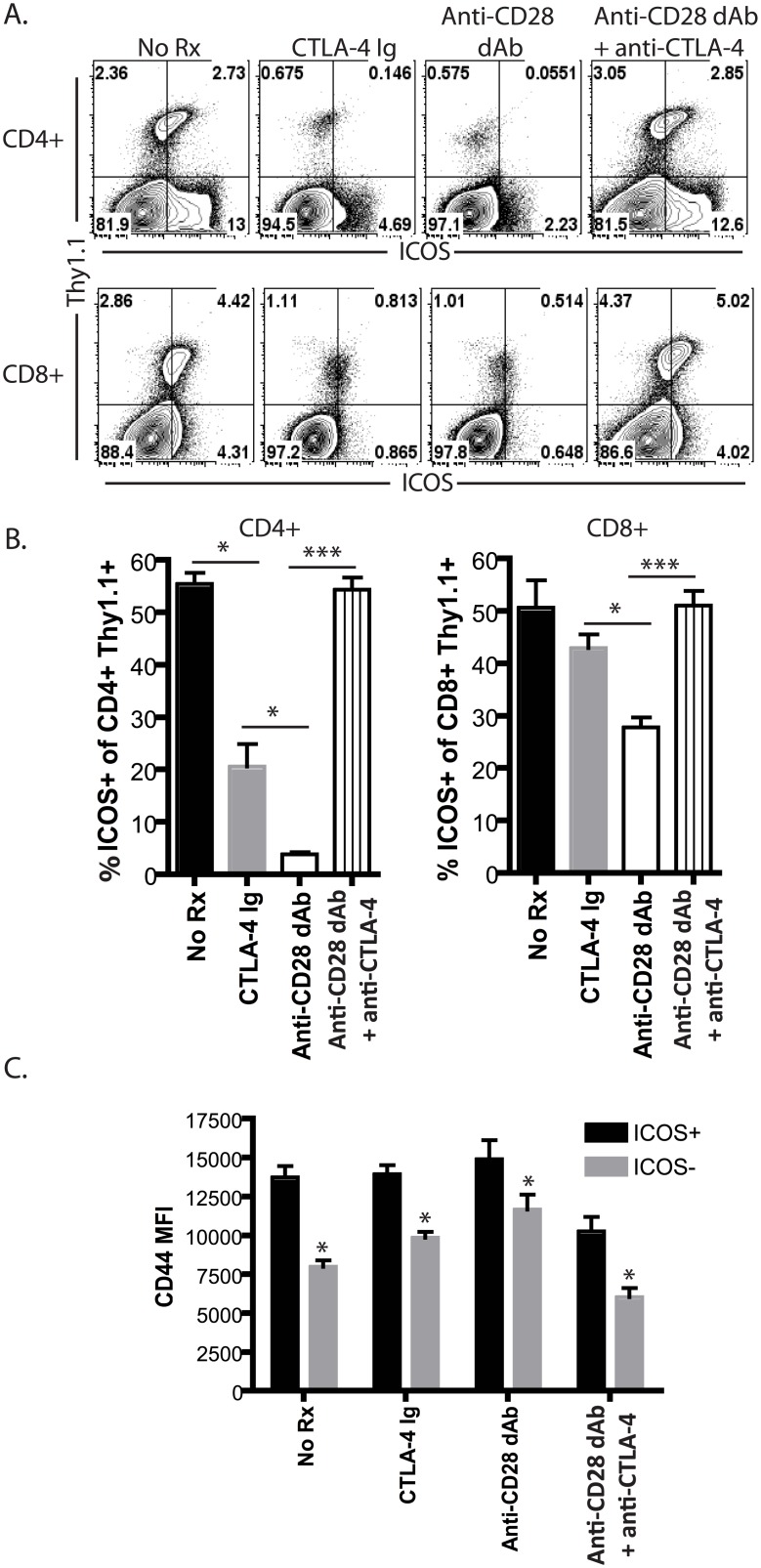
Reduced ICOS expression following selective CD28 blockade is CTLA-4 dependent. Naïve B6 animals were adoptively transferred with 10^6^ congenically labeled Thy1.1^+^ CD8^+^ OT-I T cells and 10^6^ Thy1.1^+^ CD4^+^ OT-II T cells and then challenged with an OVA-expressing skin graft. Mice remained untreated or were treated with CTLA-4 Ig, anti-CD28 dAb, or anti-CD28 dAb ^+^ anti-CTLA-4. Animals were sacrificed on day 10 post-transplant and draining LN cells were analyzed by flow cytometry. A, ICOS expression on antigen-specific Thy1.1^+^ CD4^+^ and CD8^+^ T cells on day 10 post-transplant. B, Summary data from multiple animals n = 5-8/group. C, Antigen-specific Thy1.1^+^ CD8^+^ T cells analyzed on day 10 post-transplant were gated on ICOS^+^ vs. ICOS^-^ cells, and results show CD44 expression (MFI) in these two subsets in the indicated treatment groups (n = 5-8/group). *p<0.05.

In order to determine the mechanisms underlying the observed reduced ICOS expression following selective CD28 blockade as compared to CTLA-4 Ig [15], we interrogated the requirement for preserved CTLA-4 coinhibitory signals in mediating this effect. Naïve B6 animals were adoptively transferred with 106 congenically labeled Thy1.1^+^ CD8^+^ OT-I T cells and 106 Thy1.1^+^ CD4^+^ OT-II T cells and then challenged with an OVA-expressing skin graft. Mice remained untreated or were treated with CTLA-4 Ig, anti-CD28 dAb, or anti-CD28 dAb ^+^ anti-CTLA-4 as described in the materials and methods. Animals were sacrificed on day 10 post-transplant and draining LN cells were analyzed by flow cytometry. Results showed that, as expected, ICOS was upregulated on the majority of antigen-specific CD4^+^ and CD8^+^ T cells by day 10 post-transplant (Fig 1A). As we reported previously, treatment with CTLA-4 Ig resulted in decreased ICOS expression on antigen-specific cells in both the CD4^+^ and CD8^+^ T cell compartments, but animals treated with anti-CD28 dAb experienced an even more profound reduction in ICOS expression (Fig 1B). Within each treatment group, antigen-specific CD8^+^ cells that were ICOS^+^ exhibited increased CD44 expression as compared to those that were ICOS-, indicating that under all conditions decreased ICOS expression correlated with reduced activation (Fig 1C).

In order to determine whether preserved CTLA-4 coinhibitory signals in cells isolated from animals treated with anti-CD28 dAb were responsible for the more profound reduction in ICOS expression on these cells, mice were treated with the anti-CD28 dAb in the presence of a blocking antibody to CTLA-4 as described in the materials and methods. Results indicated that when CTLA-4 signals were blocked in animals treated with selective CD28 blockade, ICOS expression levels were markedly increased relative to those treated with selective CD28 blockade alone (Fig 1A and 1B). These data thus reveal a critical role for CTLA-4 coinhibitory signaling in mediating the decreased ICOS expression observed following in vivo treatment with anti-CD28 dAb.

### Efficacy of selective CD28 blockade in controlling donor-reactive CD8^+^ T cell expansion is independent of its ability to inhibit ICOS expression

**Fig 2:**
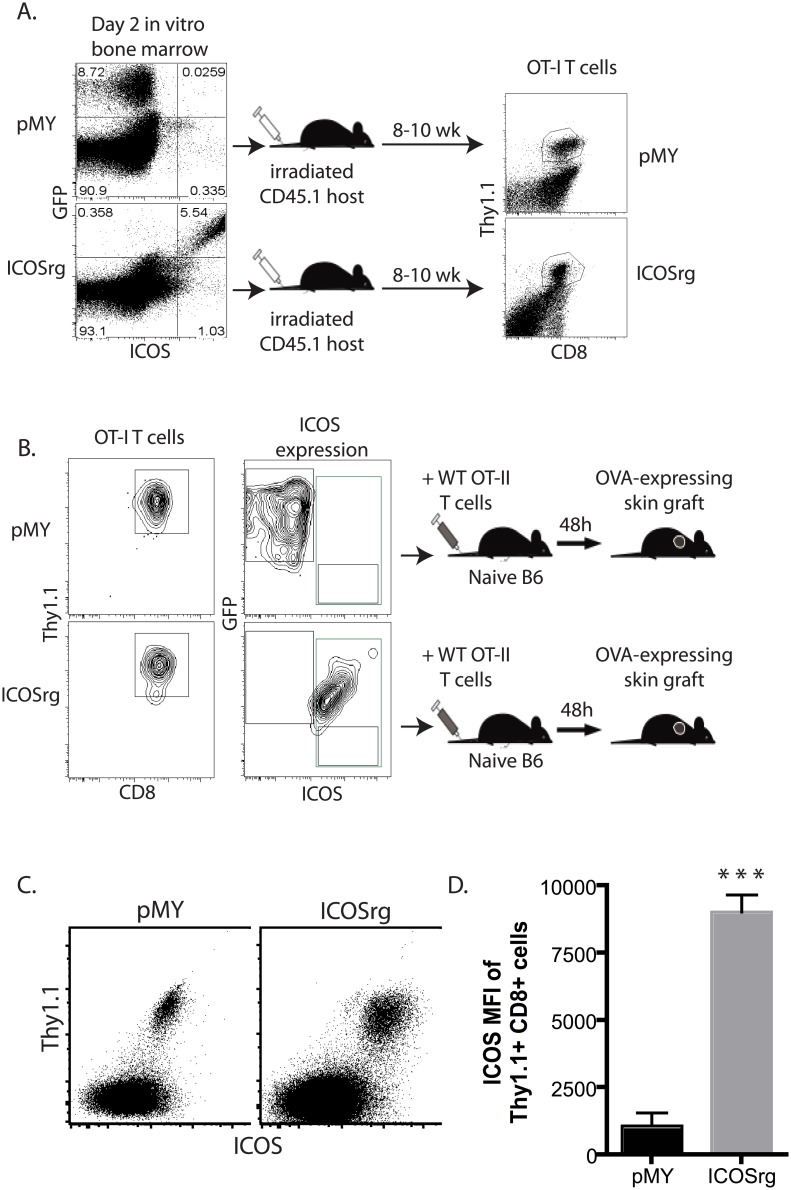
Generation of retrogenic graft-specific CD8^+^ T cells that constitutively express ICOS. To generate donor-reactive T cells that constitutively over-express ICOS, CD45.2^+^ Thy1.1^+^ OT-I bone marrow was transduced with a construct that expresses ICOS under a constitutively active viral promoter (A). The construct also contained an IRES-GFP to facilitate tracking the cells. At day 2 post transduction, ~5–10% of Thy1.1^+^ OT-I BM cells expressed either GFP alone (pMY control vector-transduced cells) or both GFP and ICOS (for ICOS vector-transduced cells). BM cells were then adoptively transferred into irradiated CD45.1^+^ Thy1.2^+^ animals. At 8–10 weeks post-in vivo transfer, Thy1.1^+^ OT-I T cells were detectable. B) GFP^+^ CD3^+^ Thy1.1^+^ (for pMY) or GFP^+^ ICOS^+^ CD3^+^ Thy1.1^+^ (for ICOSrg) OT-I T cells from spleen and LN of pMY or ICOSrg chimeric animals were FACS sorted and adoptively transferred (10^6^ /recipient) into naïve B6 hosts. Animals also received 10^6^ WT CD4^+^ Thy1.1^+^ OT-II T cells and were grafted with an OVA-expressing skin graft. C) PBL analyzed 10 days post skin grafts contained Thy1.1^+^ pMY and ICOSrg cells in the respective recipients. D) Assessment of ICOS expression on pMY vs. ICOSrg cells at day 10 post skin graft. ***p<0.0001.

In light of the observation that selective CD28 blockade and preservation of CTLA-4 mediated coinhibitory signals were associated with profoundly decreased ICOS expression, we next determined whether this reduced ICOS expression was causally related to the efficacy of selective CD28 blockade. In order to test this, we utilized a retrogenic approach to generate donor-reactive T cells that constitutively over-express ICOS, and then interrogated their expansion and function following selective CD28 blockade. Briefly, CD45.2^+^ Thy1.1^+^ OT-I bone marrow was transduced with a construct that expresses ICOS under a constitutively active promoter (Fig 2A). The construct also contained an IRES-GFP to facilitate tracking the cells. As shown in Fig 2A, ~5–10% of Thy1.1 OT-I BM cells expressed either GFP alone (pMY control vector-transduced cells) or both GFP and ICOS (for ICOS vector-transduced cells) on day 3 following transduction. BM cells were then adoptively transferred into irradiated CD45.1^+^ animals. Using this approach, in 8–10 weeks we achieved reconstitution of the T cell compartment, and were able to detect Thy1.1^+^ OT-I T cells in recipients of both pMY and ICOSrg BM (Fig 2A). We then sorted GFP^+^ CD3^+^ Thy1.1^+^ OT-I T cells from spleen and LN of pMY or ICOSrg chimeric animals (Fig 2B), and of these, all are GFP^+^ (for pMY mice) or GFP^+^ ICOS^+^ (for ICOSrg mice), indicating the ICOS and GFP transgenes are stably expressed. pMY or ICOSrg Thy1.1^+^ OT-I T cells were then adoptively transferred (106 /recipient) into naïve B6 hosts. Animals also received 106 WT CD4^+^ Thy1.1^+^ OT-II T cells and were grafted with an OVA-expressing skin graft. Analysis of peripheral blood of these recipients 10 days after transfer confirmed that both pMY and ICOSrg cells were viable (Fig 2C), and that ICOSrg cells continued to express high levels of the ICOS receptor on the cell surface (Fig 2D). The ability of our ICOS protein to signal was confirmed in separate studies which showed that expression of this construct functioned to increase CD8^+^ T cell expansion and alter memory differentiation in a model of *Listeria* infection (data not shown).

**Fig 3:**
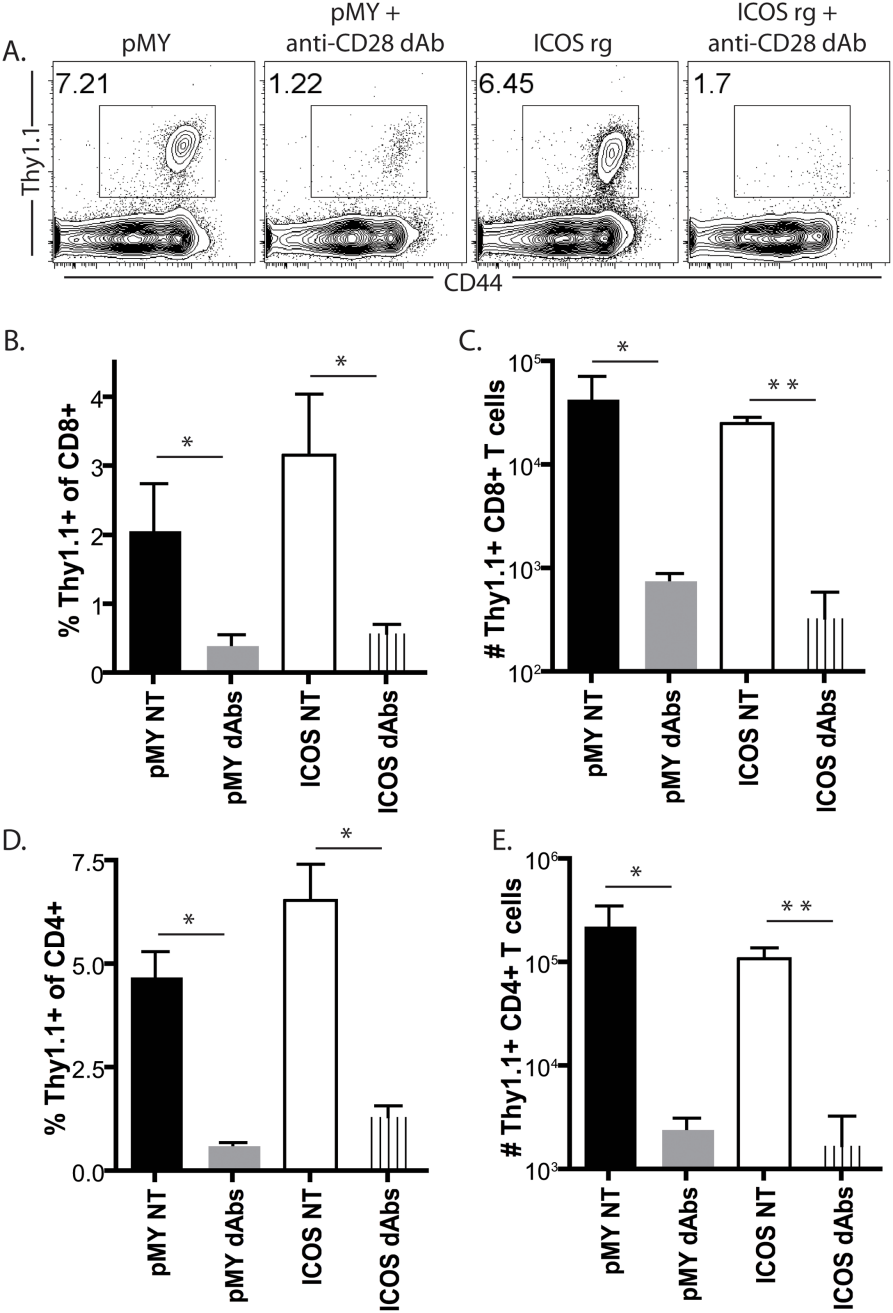
Efficacy of selective CD28 blockade in controlling donor-reactive CD8^+^ T cell expansion is independent of its ability to inhibit ICOS expression. Naïve B6 animals were adoptively transferred with 10^6^ congenically labeled ICOSrg Thy1.1^+^ OT-I T cells (or pMY Thy1.1^+^ OT-I controls) along with 10^6^ Thy1.1^+^ CD4^+^ WT OT-II T cells and then challenged with an OVA-expressing skin graft. Groups of animals were left untreated or treated with anti-CD28 dAb. Animals were sacrificed at day 10 post transplant, and graft-draining LN T cells were analyzed for the expression of the magnitude of the CD8^+^ Thy1.1^+^ response. A, Representative flow cytometry plots depicting frequencies of CD44^hi^ Thy1.1^+^ CD8^+^ T cells in the treatment groups indicated. B, Summary data of the frequencies of CD44^hi^ Thy1.1^+^ CD8^+^ T cells (three independent experiments with a total of n = 6–9 animals/group). C, Summary data of the absolute numbers of CD44^hi^ Thy1.1^+^ CD8^+^ T cells (three independent experiments with a total of n = 6–9 animals/group). D, Summary data of the frequencies of CD44^hi^ Thy1.1^+^ CD4^+^ T cells (three independent experiments with a total of n = 6–9 animals/group). C, Summary data of the absolute numbers of CD44^hi^ Thy1.1^+^ CD4^+^ T cells (three independent experiments with a total of n = 6–9 animals/group). *p<0.05, **p<0.01.

To determine the impact of constitutive ICOS expression on the efficacy of selective CD28 blockade, groups of animals were left untreated or treated with anti-CD28 dAb. Animals were sacrificed at day 10 post transplant, and splenic and graft-draining LN T cells were analyzed for the expression of the magnitude of the CD8^+^ Thy1.1^+^ response. Results revealed a detectable population of CD44hi Thy1.1^+^ CD8^+^ T cells in recipients of pMY cells (Fig 3A). Interestingly, constitutive ICOS overexpression did not impact the magnitude of donor-reactive CD8^+^ T cell expansion in the absence of immune modulation (Fig 3B and 3C). As expected, anti-CD28 dAb treatment of recipients of pMY OT-I T cells resulted in a marked inhibition of the antigen-specific CD8^+^ T cell response (Fig 3A–3C). Strikingly, however, ICOSrg graft-specific CD8^+^ T cells were similarly profoundly inhibited following selective CD28 blockade. The constitutive overexpression of ICOS on donor-reactive CD8^+^ T cells also did not alter the donor-reactive CD4^+^ T cell response, either in the presence or absence of selective CD28 blockade (Fig 3D and 3E). Overall, these results indicate that reduced ICOS expression is not the mechanism by which selective CD28 blockade more potently inhibits the expansion of donor-reactive CD8^+^ T cells following transplantation.

### Efficacy of selective CD28 blockade in inhibiting donor-reactive CD8^+^ T cell cytokine production is independent of its ability to inhibit ICOS expression

**Fig 4:**
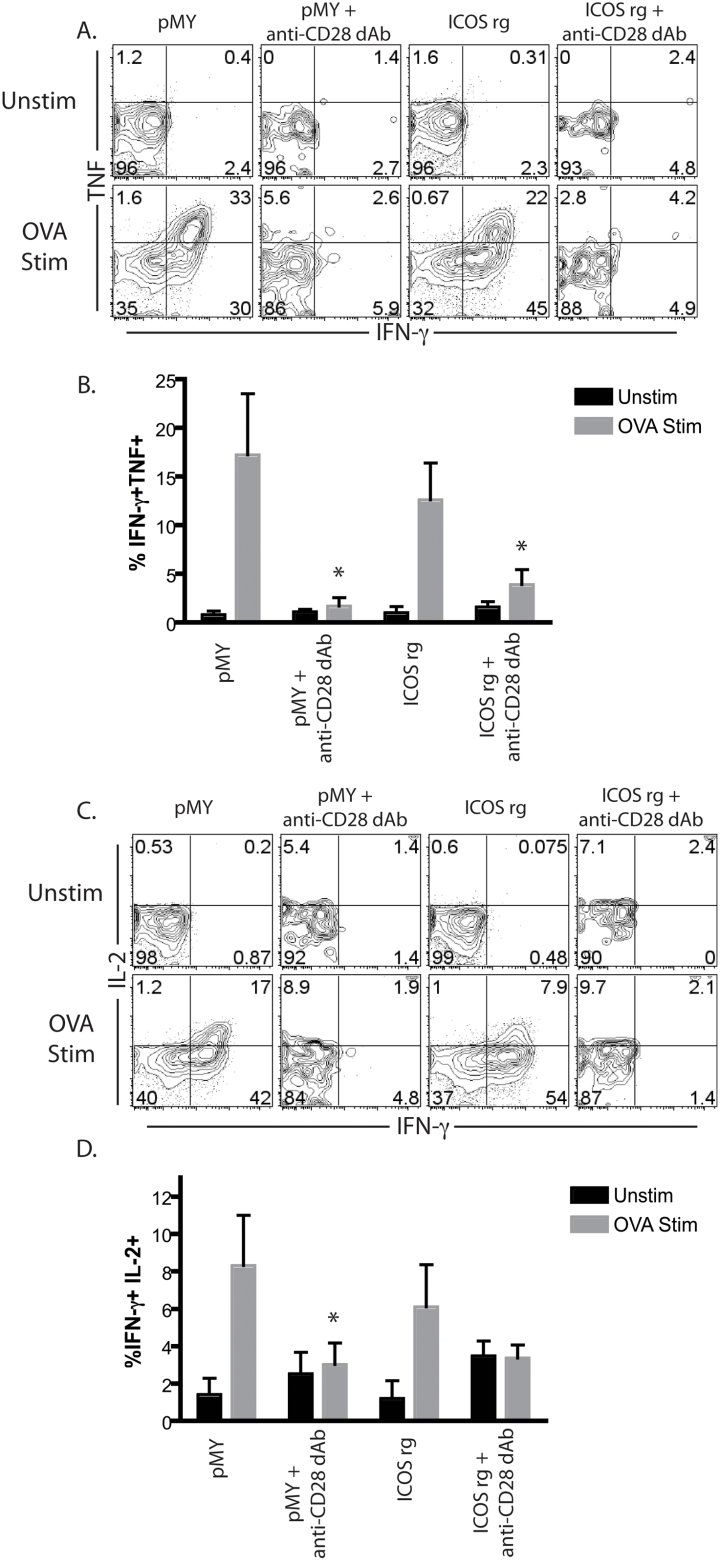
Efficacy of selective CD28 blockade in inhibiting donor-reactive CD8^+^ T cell cytokine production is independent of its ability to inhibit ICOS expression. Naïve B6 animals were adoptively transferred with 10^6^ congenically labeled ICOSrg Thy1.1^+^ OT-I T cells (or pMY Thy1.1^+^ OT-I controls) along with 10^6^ Thy1.1^+^ CD4^+^ WT OT-II T cells and were then grafted with an OVA-expressing skin graft in the presence or absence of selective CD28 blockade. Ten days post-transplant, splenocytes were restimulated for 4h in vitro with cognate SIINFEKL peptide and assessed for their ability to secrete IFN-γ, TNF, and IL-2 via intracellular cytokine staining. A, Representative flow cytometry plots depicting IFN-γ and TNF staining in the indicated treatment groups. B, Summary data of frequencies of IFN-γ^+^ TNF^+^ double producers in the indicated treatment groups (three independent experiments with a total of n = 6–9 animals/group). C, Representative flow cytometry plots depicting IFN-γ and IL-2 staining in the indicated treatment groups. B, Summary data of frequencies of IFN-γ^+^ IL-2^+^ double producers in the indicated treatment groups (three independent experiments with a total of n = 6–9 animals/group). *p<0.05.

Given the above results, we next questioned whether reduced ICOS expression was required for the ability of anti-CD28 dAb to effectively control the functionality of donor-reactive CD8^+^ T cell responses during transplantation. To test this, a similar experiment was conducted in which ICOSrg Thy1.1^+^ OT-I T cells (or pMY Thy1.1^+^ OT-I controls) were adoptively transferred into naïve B6 animals, which were then grafted with an OVA-expressing skin graft in the presence or absence of selective CD28 blockade. Ten days post-transplant, spleens and draining LN were harvested and restimulated in vitro with cognate peptide and assessed for their ability to secrete IFN-γ, TNF, and IL-2 via intracellular cytokine staining. Similar to what we observed in terms of donor-reactive CD8^+^ T cell expansion and accumulation, we observed that the constitutive overexpression of ICOS on donor-reactive T cells did not result in increased functionality in terms of their ability to secrete IFN-γ, TNF (Fig 4A and 4B), or IL-2 (Fig 4C and 4D). As expected, anti-CD28 dAb potently inhibited the frequencies of IFN-γ^+^ TNF^+^ double producers and IFN-γ^+^ IL-2^+^ double producers in recipients of pMY graft-specific OT-I T cells. Importantly, the efficacy of anti-CD28 dAb in inhibiting the acquisition of cytokine-producing effector function by donor-reactive OT-I T cells was not altered in the ICOSrg OT-I T cells (Fig 4A–4D).

### Upregulation of 2B4 (CD244) following selective CD28 blockade is independent of the degree of ICOS expression

**Fig 5:**
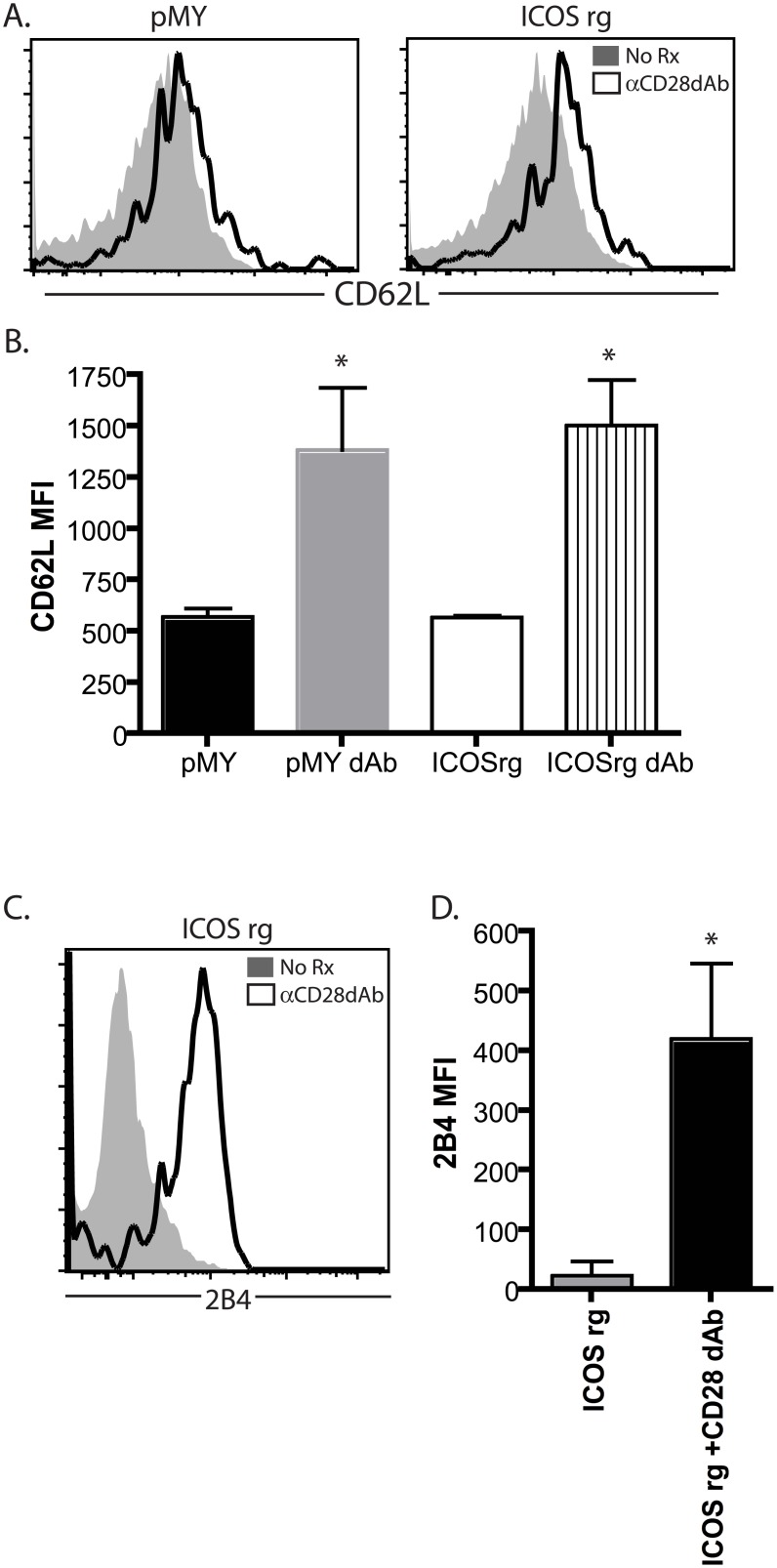
Upregulation of 2B4 (CD244) following selective CD28 blockade is independent of degree of ICOS expression. Naïve B6 animals were adoptively transferred with 10^6^ congenically labeled ICOSrg Thy1.1^+^ OT-I T cells (or pMY Thy1.1^+^ OT-I controls) along with 10^6^ Thy1.1^+^ CD4^+^ OT-II T cells and then challenged with an OVA-expressing skin graft. Groups of animals were left untreated or treated with anti-CD28 dAb. Animals were sacrificed at day 10 post transplant, and graft-draining LN T cells were analyzed for the expression of the magnitude of the CD8^+^ Thy1.1^+^ response. A, Representative flow cytometry plots depicting CD62L expression on control (left panel) or ICOSrg (right panel) Thy1.1^+^ CD8^+^ T cells in the treatment groups indicated. B, Summary data of CD62L expression (MFI) on control or ICOSrg Thy1.1^+^ CD8^+^ T cells (three independent experiments with a total of n = 6–9 animals/group). C, Representative flow cytometry plots depicting 2B4 expression on ICOSrg Thy1.1^+^ CD8^+^ T cells in the treatment groups indicated. B, Summary data of 4 expression (MFI) on ICOSrg Thy1.1^+^ CD8^+^ T cells (three independent experiments with a total of n = 6–9 animals/group). *p<0.05.

We next examined the cell surface phenotype of graft-specific pMY or ICOSrg cells in the presence or absence of selective CD28 blockade. As expected, anti-CD28 dAb treatment resulted an increase in the expression of CD62L on antigen-specific CD8^+^ T cells (Fig 5A and 5B), likely as a result of failure to downregulate CD62L during stunted activation and differentiation of these cells. Downregulation of CD62L was not rescued by overexpression of ICOS, as graft-specific ICOSrg cells similarly exhibited increased CD62L expression in the setting of anti-CD28 dAb as compared to untreated animals (Fig 5A and 5B).

We have previously reported that selective CD28 blockade is associated with upregulation of the coinhibitory molecule 2B4 on graft-specific CD8^+^ T cells [15]. 2B4 (CD244, SLAMf4) is a 38kD type I transmembrane protein and member of the CD2 subset of the immunoglobulin superfamily molecules [23, 24] that is expressed on NK cells, monocytes, basophils, and eosinophils, and is inducibly expressed on a subset of CD8^+^ T cells in both mice and humans [25–31]. In NK cells, 2B4 has been reported to have both activating and inhibitory functions [32], however recent evidence in both murine and human models indicates that its role in T cells is coinhibitory. Thus, in this set of experiments we queried whether the observed increase in 2B4 expression observed in CD8^+^ T cells in the setting of selective CD28 blockade was dependent on reduced ICOS expression. Results indicated that animals receiving ICOSrg OT-I T cells and OVA-expressing skin graft in the absence of anti-CD28 dAb did not exhibit 2B4 expression, but that in animals that were treated with anti-CD28 dAb, ICOSrg OT-I T cells markedly upregulated 2B4 (Fig 5C and 5D). These data indicate that ICOS downregulation as a result of selective CD28 blockade does not play a functional role in inducing the upregulation of the 2B4 coinhibitory molecule on these cells.

### 2B4 impacts ICOS expression in the setting of selective CD28 blockade in a cell-intrinsic manner

**Fig 6:**
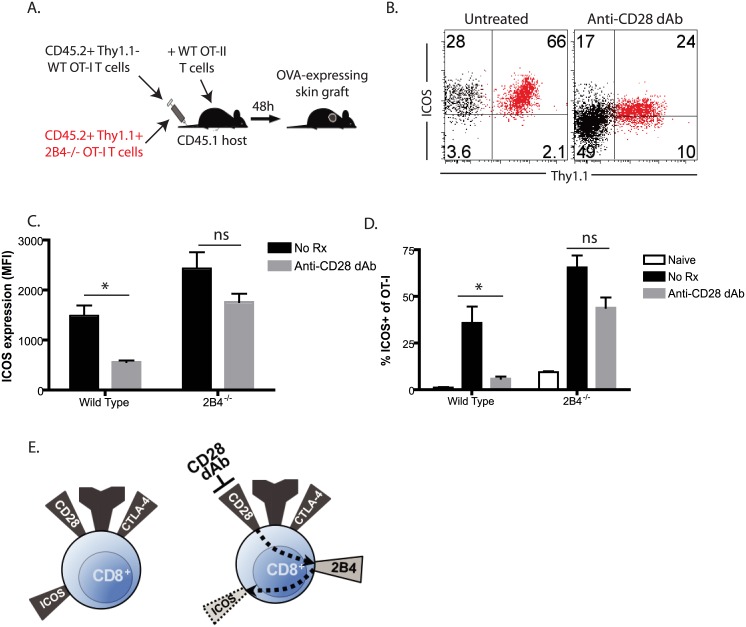
2B4 impacts ICOS expression in the setting of selective CD28 blockade in a cell-intrinsic manner. 10^6^ CD45.2^+^ Thy1.2^+^ OT-I (black) or 10^6^ CD45.2^+^ Thy1.1^+^ 2B4^-/-^ OT-I (red) were adoptively transferred along with 10^6^ CD45.2^+^ Thy1.1^+^ OT-II T cells into naïve CD45.1^+^ B6 recipients, which were then challenged with an OVA expressing skin graft in the presence of either control dAb or anti-CD28 dAb (A). Graft-draining LN were harvested on day 10 post-transplant and analyzed by flow cytometry. B, ICOS expression on WT (black, Thy1.1^-^) and 2B4^-/-^ (red, Thy1.1^+^) donor-reactive CD8^+^ Thy1.1^+^ T cells in the lymph nodes and spleens of animals treated with anti-CD28 dAb. C-D, Summary data of ICOS MFI on WT Thy1.2^+^ and 2B4^-/-^ Thy1.1^+^ T cells in the spleens on day 10 of untreated and anti-CD28 dAb treated grafted animals (C) and frequencies of WT Thy1.2^+^ ICOS^+^ and 2B4^-/-^ Thy1.1^+^ ICOS^+^ cells (D) in the spleens on day 10 of naïve (no skin graft), untreated grafted, anti-CD28 dAb treated grafted animals (two independent experiments with a total of n = 8 animals/group). E, Model of the cell intrinsic role for 2B4 in modulating ICOS expression in the setting of selective CD28 blockade. During an unmodified immune response, CD28 costimulatory signals predominate and result in ICOS expression following T cell activation (left panel). However, in the setting of CD28 dAb, 2B4 is upregulated and contribute to decreased ICOS expression in a cell-intrinsic manner. *p<0.05, ns = not significant.

Given the above findings suggesting that the lack of ICOS-mediated signals on graft-specific CD8^+^ T cells isolated from anti-CD28 dAb treated mice was not responsible for the observed increased 2B4 expression on these cells, we next interrogated the reciprocal possibility: whether 2B4 coinhibitory signals were instead functionally important for the observed reduction in ICOS signals on anti-CD28 dAb treated CD8^+^ T cell populations. In order to accomplish this, Thy1.1^+^ OT-I animals were bred onto a 2B4^-/-^ background (a kind gift of Dr. Cox Terhorst). We have previously published that 2B4 coinhibitory signals are functionally important for controlling the expansion, accumulation, and effector function of graft-specific CD8^+^ T cells during transplantation [15], and here we use a co-adoptive transfer approach to determine whether 2B4 coinhibitory signals function in a cell-intrinsic manner to limit ICOS expression in the setting of selective CD28 blockade. Briefly, Thy1.1^+^ 2B4^-/-^ T cells were co-adoptively transferred into naïve CD45.1^+^ B6 recipients along with an identical number of Thy1.2^+^ WT OT-I T cells, and animals were grafted with OVA-expressing skin grafts and treated with anti-CD28 dAb (Fig 6A). Graft-specific CD8^+^ T cell responses could be identified on the basis of their expression of CD45.2, and ICOS expression was interrogated on WT Thy1.2^+^ OT-I cells as compared to 2B4^-/-^ Thy1.1^+^ OT-I cells. In examining the WT Thy1.2 population, we observed that as expected ICOS expression was downregulated on a subset of Thy1.2^+^ WT CD8^+^ T cells isolated from both the spleens and lymph nodes of animals treated with anti-CD28 dAb (Fig 6B–6D). In contrast, ICOS was highly expressed on virtually all Thy1.1^+^ 2B4^-/-^ T cells in untreated recipients even in the presence of anti-CD28dAb (Fig 6B–6D). These results suggest that 2B4 mediated coinhibitory T cells on antigen-specific CD8^+^ T cells function in a cell-intrinsic manner to result in reduced ICOS expression observed in the setting of selective CD28 blockade.

Taken together, our results suggest a model in which selective CD28 blockade results in 2B4 upregulation on primary effectors during transplantation, and that these 2B4 coinhibitory signals function to secondarily downmodulate ICOS expression to further limit the allospecific immune response (Fig 6E). Using our co-adoptive transfer approach we were able to demonstrate that these 2B4 mediated coinhibitory signals were required in a cell-intrinsic manner, in that WT OT-I T cells exhibited reduced ICOS expression following treatment with anti-CD28 dAb, while 2B4^-/-^ OT-I adoptively transferred into the same host did not.

## Discussion

In this study we addressed the role of modulation of the ICOS pathway following selective CD28 blockade. Our previous results had indicated that CD28 blockade using novel domain antibodies resulted in upregulation of the 2B4 coinhibitory molecule and reduced expression of the ICOS costimulator [15]. Here, using ICOS retrogenic cells to induce constitutive expression of ICOS on donor-reactive CD8^+^ T cells in CD28 dAb-treated animals, we determined that reduced ICOS expression is not the mechanism by which anti-CD28 dAb mediates its inhibitory effects on these cells, in that the donor-reactive CD8^+^ T cell response was similarly attenuated by anti-CD28 dAb even in the presence of constitutive ICOS expression.

To our knowledge this study is the first to assess the effects of constitutive expression of ICOS during a primary CD8^+^ T cell response in vivo. Interestingly, we observed that constitutive ICOS overexpression did not impact the magnitude of the peak donor-reactive CD8^+^ T cell response; in that CD8^+^ Thy1.1^+^ frequencies were similar in both vector control and ICOS retrogenic populations. This finding is consistent with previous reports in the literature demonstrating that accumulation of activated effectors at the peak of the primary CD8^+^ T cell response to influenza virus infection was not altered in ICOS^-/-^ animals [33]. Other reports have found that ICOS can enhance T cell proliferation and accumulation [34], but to a lesser extent compared with CD28, presumably due to the limited amount of IL-2 produced following ICOS ligation as compared to CD28 ligation [35, 36]. It has been purported that the fact that the ability of ICOS to promote proliferation is not readily appreciable during in vivo immune responses is the result of the dominance of the CD28 costimulatory pathway [37, 38]. Here, we interrogated the role of ICOS signaling in a setting in which CD28 costimulation was pharmacologically blocked, and found that even under these conditions, forced ICOS overexpression failed to augment donor-reactive CD8^+^ T cell responses. Possible mechanisms underlying this effect include limited ligand availability. Unlike CD80/CD86, which are primarily restricted to APC, ICOS-L can be expressed on parenchymal cells [36, 39] and is upregulated in the presence of inflammatory cytokines such as TNF [36]. Thus, we would presume that under inflammatory conditions such as those present during skin transplantation, availability of ICOS-L would not be a limiting factor, however this possibility warrants further investigation. A second possible mechanism underlying this effect is the assertion that ICOS costimulation actually requires the presence of exogenous IL-2 [35], something that would be limited under conditions of CD28 blockade.

We have previously observed that 2B4^-/-^ CD8^+^ T cells fail to downregulate ICOS [15]. However, the mechanisms underlying this effect were unclear. We hypothesized that the observed reduction in ICOS expression on anti-CD28 dAb-treated cells that upregulate 2B4 could be due to cell intrinsic coinhibitory signaling. Alternatively, the fact that 2B4^-/-^ donor-reactive CD8^+^ T cells secrete increased inflammatory cytokines (including IFN-γ and TNF as we previously reported [15]) could secondarily lead to increased ICOS expression. In order to differentiate between these possibilities, in the current study we performed a co-adoptive transfer experiment where 2B4^-/-^ donor-reactive CD8^+^ T cells were co-adoptively transferred along with WT Thy1.2^+^ donor-reactive CD8^+^ T cells, and observed that in the setting of CD28 blockade, 2B4^-/-^ cells maintained high ICOS expression while WT CD8^+^ donor-reactive T cells exhibited decreased ICOS expression. From these data we conclude that it is not some trans-acting factor secreted by 2B4^-/-^ CD8^+^ T cells acting in a cell-extrinsic fashion to drive the continued expression of ICOS on these cells in the setting of CD28 antagonism, but rather the absence of a cell-intrinsic 2B4 coinhibitory signaling mechanism that results in high ICOS expression even in the absence of CD28 signaling. Interestingly, previous studies have implicated CTLA-4 coinhibitory signals as attenuating ICOS expression on T cells [35]; this study has therefore identified a second coinhibitory molecule that functions to limit ICOS expression in vivo. These findings further support the paradigm that the initial “first wave” of costimulatory and coinhibitory signals critically impact the subsequent expression of inducible cosignaling molecules in order to further fine-tune the response.

Our data also demonstrate that the observed reduced expression of ICOS in anti-CD28 dAb mice as compared to CTLA-4 Ig treated animals is due at least in part to preserved CTLA-4 signaling. These results are consistent with early studies by Riley et al. showing that ICOS expression can be blunted by CTLA-4 engagement [35], and more recently studies by Allison’s group have showed that CTLA-4 blockade combined with a tumor cell vaccine engineered to express ICOS ligand significantly enhanced tumor-specific CD8^+^ T cell responses and improve tumor clearance in a B16 melanoma model [40]. Consistent with these findings, our data revealed that anti-CD28 dAb ^+^ anti-CTLA-4 treatment resulted in a full restoration of ICOS expression similar to that observed in untreated animals, suggesting that CTLA-4 signaling results in reduced ICOS expression in the absence of CD28 signals. Interestingly, however, we observed increased ICOS expression on CTLA-4 Ig treated cells as compared to anti-CD28 ^+^ anti-CTLA-4 treated cells, both of which should theoretically have both the CD28 and CTLA-4 molecules blocked. We speculate that a possible explanation for this observation is that some CTLA-4 signals are still transmitted during treatment with CTLA-4 Ig, because the CTLA-4 Ig fusion protein may ineffectively compete with endogenous cell-bound CTLA-4 for access to CD80/CD86 ligands in vivo.

Our study interrogated the role of ICOS overexpression solely on donor-reactive CD8^+^ T cells. We chose to focus on CD8^+^ T cells because 1) previous studies have identified CD8^+^ T cells as being the primary mediators of costimulation blockade-resistant rejection [4, 22], and 2) we were interested in study the cross-regulation between 2B4 and ICOS, and our previous publication had identified 2B4 as being upregulated only on CD8^+^ T cells in the setting of CD28 blockade and not on CD4^+^ T cells [15]. Our data therefore do not exclude a potential role for reduced ICOS expression on CD4^+^ T cells as contributing to the observed increased efficacy of anti-CD28 dAb. Indeed, ICOS is known to play an important role in CD4^+^ T cell responses [41], particularly in terms of the provision of help for B cells [7, 42–44], and thus future investigation into the role of ICOS on CD4^+^ T cells in the setting of costimulation blockade is warranted. Along these lines, while by every metric we have assessed including expansion, differentiation, and cytokine secretion, ICOS overexpression had no impact on the graft-specific CD8^+^ T cell response in the setting of CD28 blockade, we cannot rule out that ICOS overexpression could have an impact on allograft rejection by altering the response in some other manner.

Combined with our previous publication demonstrating the functional role of 2B4 upregulation on graft-specific CD8^+^ T cells in the enhanced efficacy of anti-CD28 dAb as compared to CTLA-4 Ig [15], the data presented in this study put forth a model of CD28 dAb inhibition in which blockade of CD28 costimulatory signals in the presence of preserved coinhibitory CTLA-4 signals results in 2B4 upregulation, which subsequently leads to a decrease in ICOS expression on donor-reactive CD8^+^ T cells. These findings are of potential clinical significance in that CD28 negative cells exist at significant frequencies in immunologically experienced humans and thus have been purported to be a barrier for CD28 blockade-induced long-term graft survival [45–48]. There is considerable interest in identifying additional costimulatory pathways that may control the recall responses in these highly differentiated cell populations. Our finding that constitutive overexpression of ICOS in the absence of CD28-mediated signals failed to impact donor-specific CD8^+^ T cell responses suggests that targeting ICOS may not be an effective strategy to inhibit these cells. This idea is supported by data showing that ICOS blockade failed to impact belatacept-resistant rejection in non-human primate model of renal transplant rejection [49]. Identification of costimulatory pathways that impact recall responses of CD28^-^ memory T cells in the setting of transplantation remains an important goal.
